# Multimodal assessment of sleep-wake perception in insomnia disorder

**DOI:** 10.1038/s41598-025-00995-3

**Published:** 2025-06-05

**Authors:** Carlotta L. Schneider, Kristoffer D. Fehér, Elisabeth Hertenstein, Fabian Hügli, Marina Wunderlin, Marc A. Züst, Christian Mikutta, Antoine R. Adamantidis, Thomas Berger, Dieter Riemann, Bernd Feige, Christoph Nissen

**Affiliations:** 1 Department of Psychiatry, Faculty of Medicine, University of Geneva, Geneva, Switzerland; 2 University Hospital of Psychiatry and Psychotherapy, University of Bern, Bern, Switzerland; 3 Graduate School for Health Sciences, University of Bern, Bern, Switzerland; 4 University Hospital of Old Age Psychiatry and Psychotherapy, University of Bern, Bern, Switzerland; 5 Privatklinik Meiringen, Meiringen, Switzerland; 6 Center for Experimental Neurology, Department of Neurology, University Hospital Bern, Sleep-Wake Epilepsy Center, Inselspital, University of Bern, NeuroTec, Bern, Switzerland; 7 Department of Clinical Psychology and Psychotherapy, University of Bern, Bern, Switzerland; 8 Department of Psychiatry and Psychotherapy, Faculty of Medicine, Medical Center - University of Freiburg, University of Freiburg, Freiburg, Germany; 9 Department of Psychiatry, Geneva University Hospitals (HUG), Geneva, Switzerland

**Keywords:** Insomnia disorder, Sleep-wake perception, Polysomnography, Spectral analysis, Serial awakening, Sleep, Psychiatric disorders

## Abstract

Insomnia disorder is a prevalent health problem. The primary diagnostic criterion is a subjective complaint about reduced quantity or quality of sleep, which is often not observed in objective sleep measurements. Here we show that patients with insomnia disorder, characterized by substantial subjective sleep complaints, did not differ on objective measures of sleep continuity, sleep architecture, spectral power, spectral slope, and phase-amplitude coupling of slow oscillatory and spindle activity. Perception of wakefulness following serial awakenings from NREM sleep was frequent in both patients and controls, with no significant group difference. High frequency spectral power, as an index of cortical arousal prior to awakening, but not standard measures of sleep, predicted the perception of wakefulness across groups, possibly related to physiological wake-like activity during sleep. Our results support the notion that sleep-wake regulatory systems and direct sleep-wake perception are often intact in patients with insomnia disorder. These results propose empirical support for cognitive behavioral therapy for insomnia as the first-line treatment.

## Introduction

Insomnia disorder is a frequent health problem. According to current diagnostic systems^1−3^ the primary criterion is a subjective complaint of poor sleep, which is often not observed in objective sleep measurements. This discrepancy limits the understanding of the pathophysiology and new treatment developments.

Insomnia disorder is prevalent, affecting around 5 to 10% of the adult population^4^. Diagnostic systems define insomnia disorder as a predominant complaint of dissatisfaction with sleep quantity or quality across several nights per week over a period of at least 3 months and related subjective daytime distress^1–3^. Besides compromising quality of life, insomnia disorder is associated with adverse health outcomes, such as the risk of developing major depression^5^. The current first-line treatment is Cognitive Behavioral Therapy for Insomnia (CBT-I) with good efficacy; still, 30% of patients do not experience full remission^4^. Pharmacotherapy can be offered for short-term treatment, but is associated with the risk of side-effects, tolerance and dependence, indicating the need for novel concepts and treatments.

A core enigma in sleep research and a fundamental limitation for advancement in clinical care is a poor understanding of sleep-wake perception^6,7^. Patients with insomnia disorder often report a substantial decrease in subjective total sleep time in contrast to a minor reduction in objective (polysomnographic) total sleep time^8^. In the absence of animal models and direct perception measures during sleep in humans, reports on the pre-awakening state through serial awakenings represent the best index of sleep-wake perception. Studies in healthy individuals have noted instances of subjective wakefulness after awakenings out of non-rapid eye movements (NREM) and rapid eye movement (REM) sleep, ranging between 38 and 56% and 21–26%, respectively^9,10^. Some studies on insomnia disorder observed more frequent wake reports after awakenings from NREM sleep in patients compared to controls^11–13^, whereas others did not^14,15^. Some studies observed more wake reports from REM sleep in patients compared to controls^12,13,15^, whereas others did not^14^. The proportion of wake reports ranged between 12 and 96% (NREM sleep) and 0.8–65% (REM sleep) in patients and controls, potentially related to differences in samples and protocols. Together, the perception of wake and sleep and its neural underpinnings need to be further determined.

A potential explanation for wake reports from polysomnographic sleep derives from concepts of fluid sleep-wake regulation. Studies in animals^16^ and humans^17^ converge that sleep and wake activity patterns are not mutually exclusive but can coexist with ambiguous boundaries^18^. Our study aimed to further investigate parameters of sleep-wake regulation beyond polysomnography, including high frequency (beta) spectral power as a marker of cortical arousal^19^, slow wave count and amplitude, spectral slope as an index of non-oscillatory brain activity related to arousal levels^20^, and phase-amplitude coupling of sleep slow oscillatory and spindle activity in thalamo-cortical circuits^21^.

The current study aimed to further characterize sleep and sleep-wake perception in patients with insomnia disorder and healthy controls through serial awakenings from NREM sleep. We hypothesized that patients with a high level of subjective sleep complaint show indices of disrupted sleep at baseline (polysomnography, spectral power, spectral slope, and phase amplitude coupling), and perceive NREM sleep more often as wake than controls. Further we explored potential neural correlates of sleep-wake perception.

## Results

Thirty patients with insomnia disorder (20 female, 8 male, 2 diverse, 39 ± 15 years) and 30 healthy controls (21 female, 9 male, 36 ± 13 years) were characterized clinically and tested across 3 nights in the sleep laboratory. After an adaptation and baseline night, a total of 559 awakenings (up to 12 per participant) were performed from non-rapid eye movement (NREM) sleep in night 3. Participants reported on their sleep-wake perception through an automatized interview.

### Demographic and clinical characteristics

As shown in Table 1, healthy controls and patients with insomnia disorder had similar demographic characteristics. Patients suffered from insomnia disorder for 15.7 years on average. Patients did not report increased sleepiness compared to healthy controls, as measured by the Epworth sleepiness scale (ESS)^22^. Patients reported worse subjective sleep on the Insomnia severity index (ISI)^23^, worse sleep quality and habits on the Pittsburgh sleep quality index (PSQI)^24^, lower perception of general health as measured by the Short form health survey (SF-36)^25^, higher scores on the Glasgow sleep effort scale (GSES)^26^ measuring the persistent preoccupation with sleep, higher levels of fatigue measured by the Multidimensional fatigue inventory (MFI)^27^, more dysfunctional beliefs and attitudes about sleep on the Dysfunctional beliefs and attitudes about sleep scale (DBAS)^28^ and higher scores on cognitive and somatic arousal experienced at bedtime on the pre-sleep arousal scale (PSAS)^29^. All questionnaires were filled-out before the first sleep laboratory night.

**Table 1:** Demographic and clinical characteristics.

	Healthy controls (*n* = 30)	Patients with insomnia disorder (*n* = 30)	*p*-value	Effect size
Sex	21 f, 9 m	20 f, 8 m, 2 d	*p* = 0.353^1^	0.18
Age (years)	36.1 ± 12.5 (18–65)	38.5 ± 15.4 (19–65)	*p* = 0.510	0.17
Years of education	16.0 ± 3.0	16.1 ± 3.2	*p* = 0.934	0.02
Duration of insomnia disorder (years)	0.0 ± 0.0	15.7 ± 14.4	–	-
ESS	6.9 ± 3.4	6.1 ± 3.1	*p* = 0.324	0.26
ISI	3.6 ± 2.7	11.8 ± 3.7	*p* < 0.001	2.50
PSQI	3.1 ± 1.6	8.3 ± 2.7	*p* < 0.001	2.35
SF-36 general health	77.9 ± 9.4	65.5 ± 12.6	*p* < 0.001	1.02
GSES	1.9 ± 1.6	6.5 ± 2.8	*p* < 0.001	2.01
MFI	33.1 ± 10.8	48.7 ± 14.8	*p* < 0.001	1.21
DBAS	2.9 ± 1.0	4.4 ± 1.7	*p* < 0.001	1.06
PSAS cognitive	14.4 ± 4.2	22.4 ± 7.1	*p* < 0.001	1.37
PSAS somatic	10.0 ± 2.0	13.0 ± 3.6	*p* < 0.001	1.02

Data represent means ± standard deviations, or frequencies for categorical data. Cohen’s *d* is reported as an estimate of effect size. f, female. m, male. d, diverse. Independent sample t-tests, except^1^Chi square test. Significant p-values are shown in bold. ESS, Epworth sleepiness scale; ISI, Insomnia severity index; PSQI, Pittsburgh sleep quality index; SF-36, Short form health survey, general health perception; GSES, Glasgow sleep effort scale; MFI, Multidimensional fatigue inventory; DBAS, Dysfunctional beliefs and attitudes about sleep; PSAS, Pre-sleep arousal scale, cognitive and somatic.

Patients were not pre-selected or categorized depending on a potential discrepancy between subjective and objective total sleep time. From the baseline data, a sleep perception index was calculated for both groups (subjective TST/objective TST*100)^13^, with 101.2$\:\pm\:$14.6% in patients and 108.7$\:\pm\:$13.4%) in controls (*p*=0.046, Cohen’s *d* = 0.530). A chi-square test showed that there were significantly more participants who underestimated their TST (sleep perception index < 100%) in the patient (*n*=13) compared to the control group (*n*=5; *p*=0.019, Cohen’s *d* = 0.630). Of note, none of the participants had a sleep perception index < 60%, which has previously been used as a cut-off for categorizing “misperceptors”^13^.

## Polysomnography

**Fig. 1:**
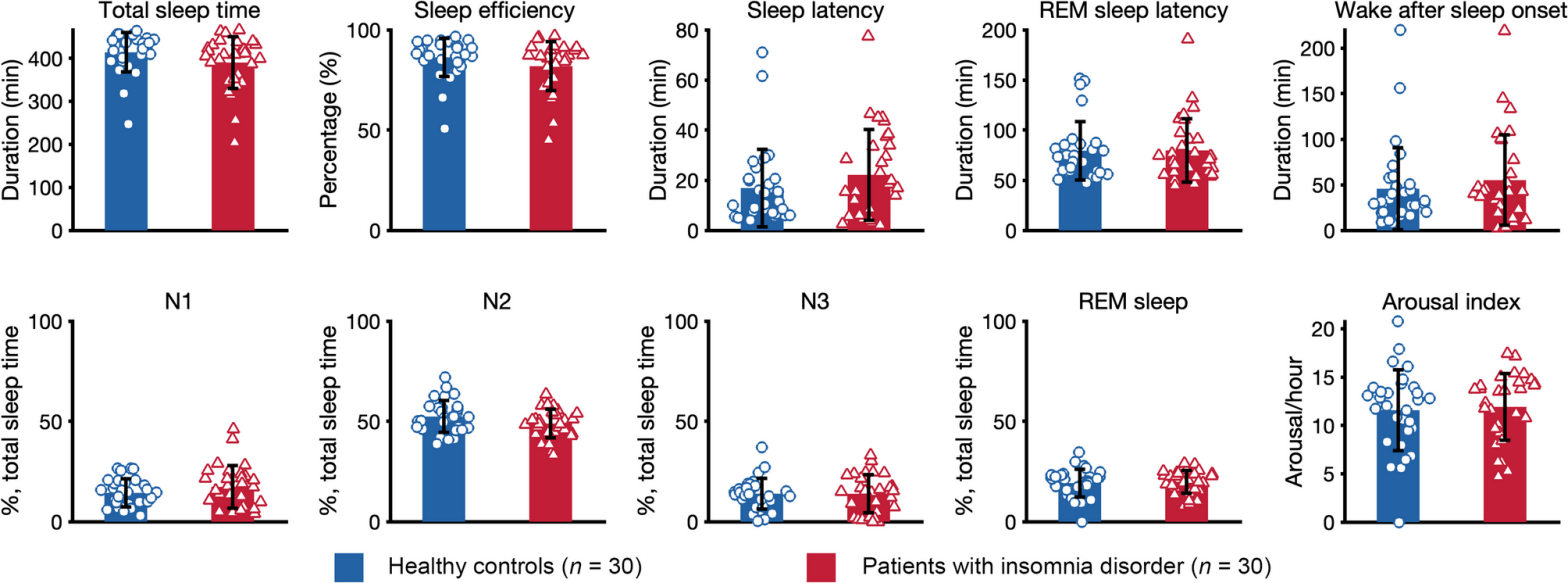
Sleep continuity and architecture. The figure depicts polysomnographic parameters of the baseline night for healthy controls and patients with insomnia disorder. T-test for independent samples did not reveal any group difference (all *p* > 0.05).

As depicted in Fig. 1 and Supplementary Table 1, patients did not differ from controls on polysomnographic parameters in the baseline night. The data shows similar durations for total sleep time, sleep latency, REM sleep latency, and wake after sleep onset (represented in min), and similar sleep efficiency, N1 sleep, N2 sleep, N3 sleep, REM sleep (percentage of total sleep time), and arousal index (number of arousals per hour) between the groups.

## Sleep microstructure

**Fig. 2:**
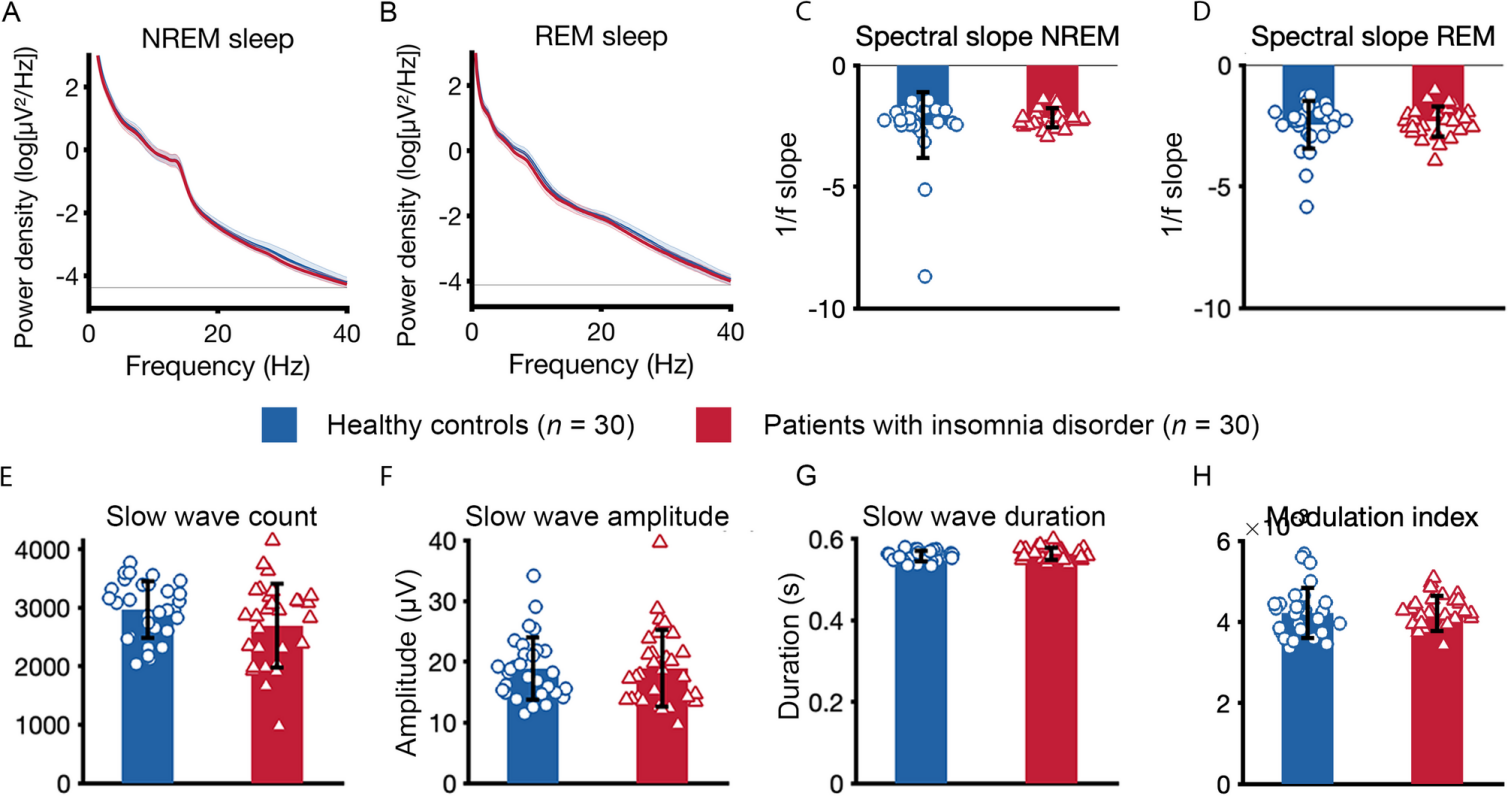
Sleep microstructure. The figure shows parameters of sleep microstructure of the baseline night for healthy controls and patients with insomnia disorder (calculated as an average across the scalp). (**A**) Log transformed power spectral density during NREM sleep (95% CI). (**B**). Log transformed power spectral density during REM sleep (95% CI). (**C**) Spectral slope NREM sleep. (**D**) Spectral slope REM sleep. (**E**) Slow wave count. (**F**) Slow wave amplitude. (**G**) Slow wave duration. (**H**) Modulation index during N2 and N3 sleep.

Patients with insomnia disorder did not differ from healthy controls on spectral power values during NREM sleep (Fig. 2A) or REM sleep during the baseline night (Fig. 2B). Furthermore, patients did not differ from controls on other microstructure parameters, including spectral slope in NREM (Fig. 2C) and REM sleep (Fig. 2D), slow wave count (Fig. 2E), slow wave amplitude (Fig. 2F), slow wave duration (Fig. 2G) and modulation index (Fig. 2H).

## Topographic analysis

**Fig. 3:**
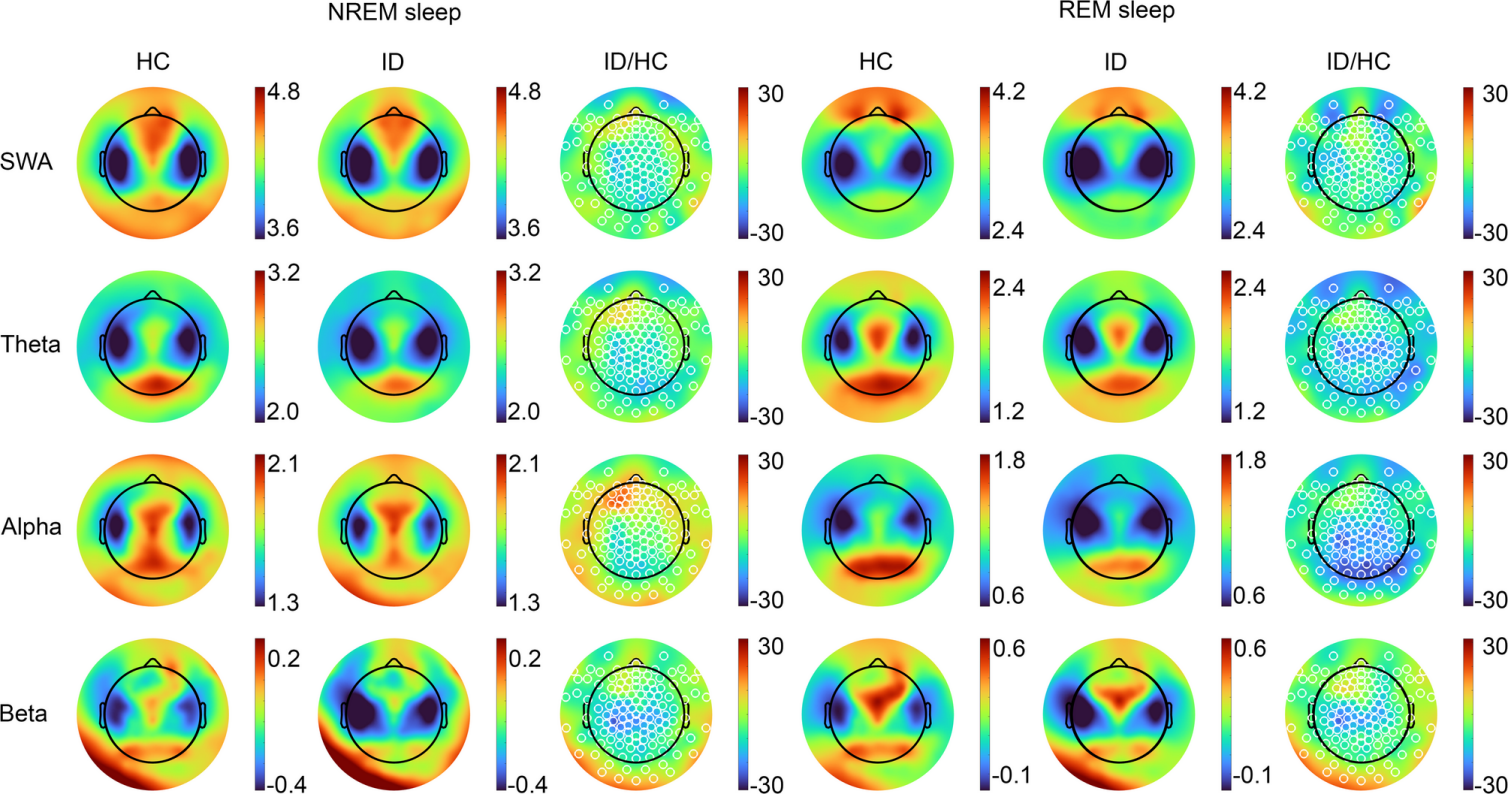
Spectral power densities across channels. The figure shows topographic distributions across derivations of spectral power density across frequency bands (log(µV^2^), during NREM (columns 1–3) and REM sleep (columns 4–6), for healthy controls (HC) and patients with insomnia disorder (ID). Columns 3 and 6 (ID/HC) depict the differences in percentage between patients with insomnia disorder and healthy controls (percentages based on non-log transformed spectral power density values). SWA, slow wave activity. Channel-wise independent samples t-tests (FDR corrected across channels) on log transformed spectral power density did not reveal any group difference (all *p* > 0.05).

Figure 3 depicts spectral power densities across the scalp during NREM and REM sleep. Channel-wise comparisons did not reveal any significant group differences for regional power values (all *p* > 0.05; independent samples t-test; corrected for multiple comparisons across channels by false discovery rate^30^.

## Subjective sleep parameters

Patients, compared to controls, reported significantly reduced subjective total sleep time, sleep efficiency, sleep quality and heightened sleep latency in the morning report (sleep diary) following the baseline night. Furthermore, they reported increased daytime tiredness, worse concentration and mood, and a reduced feeling of restoration compared to controls (Supplementary Table 1). This analysis suggests that the baseline night can be considered as representative for subjective perception.

### Sleep-wake perception

**Fig. 4:**
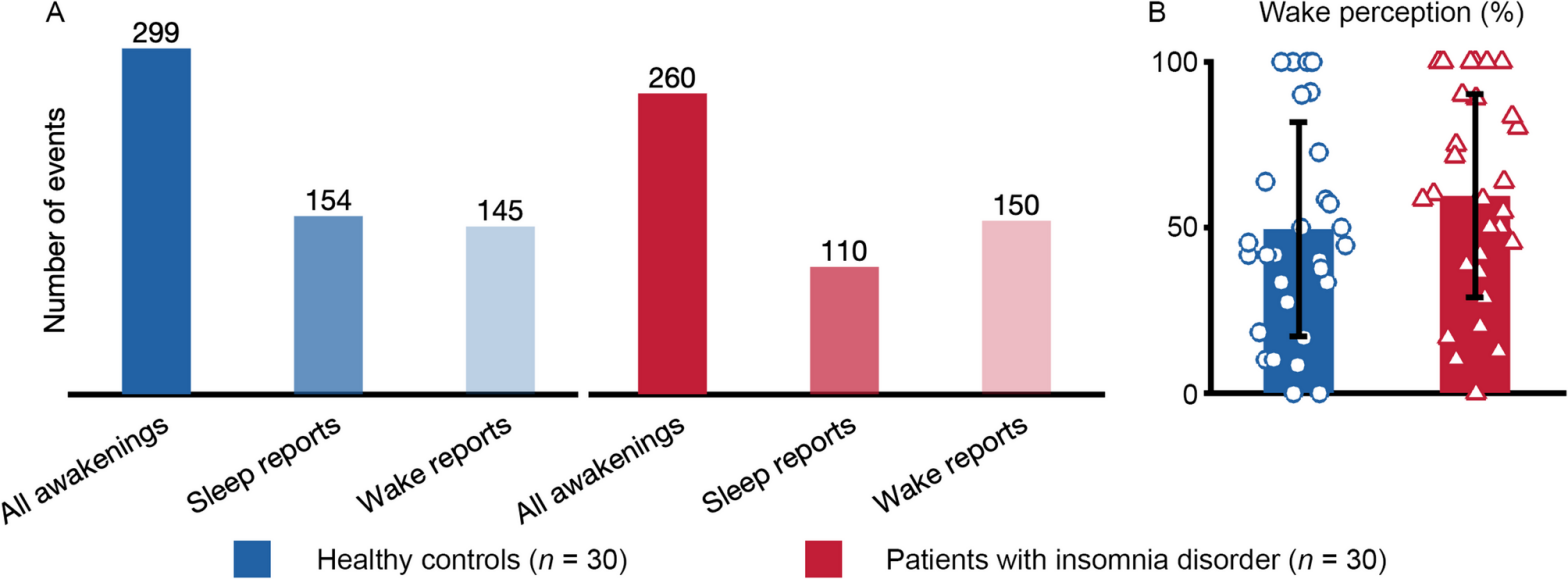
Sleep-wake perception. The figure visualizes results from a total of 559 serial awakening reports of healthy controls and patients with insomnia disorder. (**A**) A total of 299 awakenings in healthy controls with 154 sleep and 145 wake reports. A total of 260 awakenings in patients with insomnia with 110 sleep and 150 wake reports. (**B**) Percentage of wake reports after awakening from NREM sleep, for healthy controls (49.4 ± 32.3%) and patients with insomnia disorder (59.4 ± 30.7%). A t-test for independent samples did not reveal a significant group difference (*p* = 0.223; Cohen’s d = 0.32, small effect size).

A total of 559 successful awakenings from NREM sleep were performed (299 in controls, 260 in patients), with 154 sleep versus 145 wake reports in controls (Fig. 4A), and 110 sleep versus 150 wake reports in patients (Fig. 4A). There was no significant difference in the percentage of wake reports between controls (49.4 ± 32.3%) and patients (59.4 ± 30.7%, t-test for independent samples, *p* = 0.223; Cohen’s d = 0.32 (small effect size), Fig. 4B).

The selected awakenings represent a selection of “successful events” out of a total of 893 awakenings. Successful events were defined by a deliberate awakening from stable NREM sleep (2 min, that is 4 epochs of NREM sleep prior to the awakening epoch, validated offline) in combination with a response to the awakening signal and a valid answer to at least the first interview question. From 893 awakenings, 79 events were excluded due to PSG wake or REM sleep during the 2 min PSG block. 225 were excluded due to non-response from the participants (103 in healthy controls and 122 in patients with insomnia) and 30 events were excluded due to invalid response to the interview questions.

## Control parameters

**Fig. 5:**
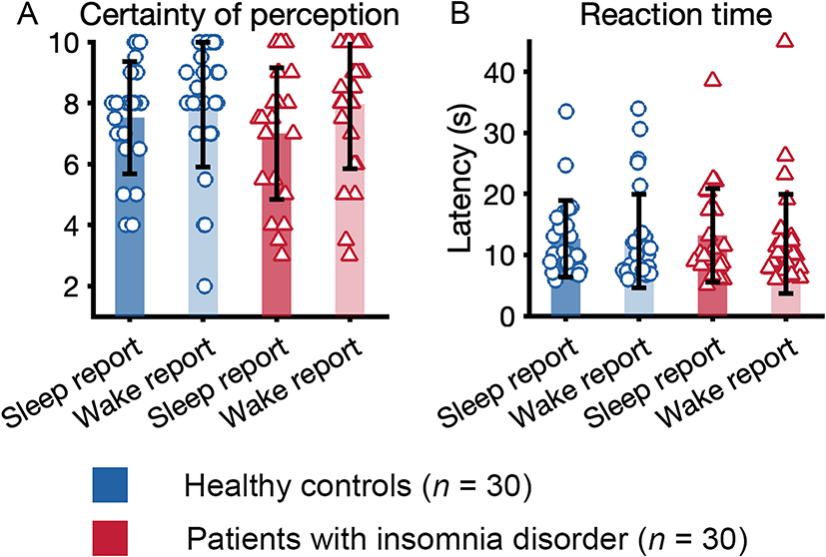
Control parameters. (**A**) Certainty of sleep or wake perception across groups and perception. Certainty was assessed on a scale of 1–10, 10 being very certain. (**B**) Reaction time, measured from awakening signal (vibration bracelet) to indication of wakefulness (microswitch) across groups and perception.

To further understand the quality of the perception we analyzed the certainty of the answer to question 1 and the reaction time between awakening signal and indication of awakening by the participant (Fig. 5). The analysis of certainty (“How certain were you, from 1 to 10”, 1 being very uncertain and 10 being very certain) in their answer to the first question (“Just now, were you awake or asleep?”) demonstrated a high certainty without any significant differences between group or condition (all *p* > 0.05). Furthermore, Reaction time to the awakening signal showed no differences between group or condition (all *p* > 0.05) confirming that the duration of the awakening procedure was not predictive of perception.

## Sleep prior to awakenings

By definition, a successful awakening was preceded by NREM sleep, excluding REM sleep or wake. Sleep prior to successful awakenings included N1 (1.2%$\:\pm\:$5.0% in controls, 9.2%$\:\pm\:$18.0% in patients), N2 (90.8%$\:\pm\:$14.7% in controls, 69.6%$\:\pm\:$32.6 in patients) and N3 sleep (5.4%$\:\pm\:$13.4% in controls, 17.9%$\:\pm\:$34.5% in patients). ANOVAs indicated significant effects for the factor group (patients/controls) for sleep stages N1 (post-hoc more N1 in patients) and N2 (post-hoc less N2 in patients), but not N3, and no significant effect for the repeated measures factor sleep-wake perception (sleep/wake) and no significant interaction effect. This lack of interaction indicates that PSG parameters did not predict sleep-wake perception.

**Fig. 6:**
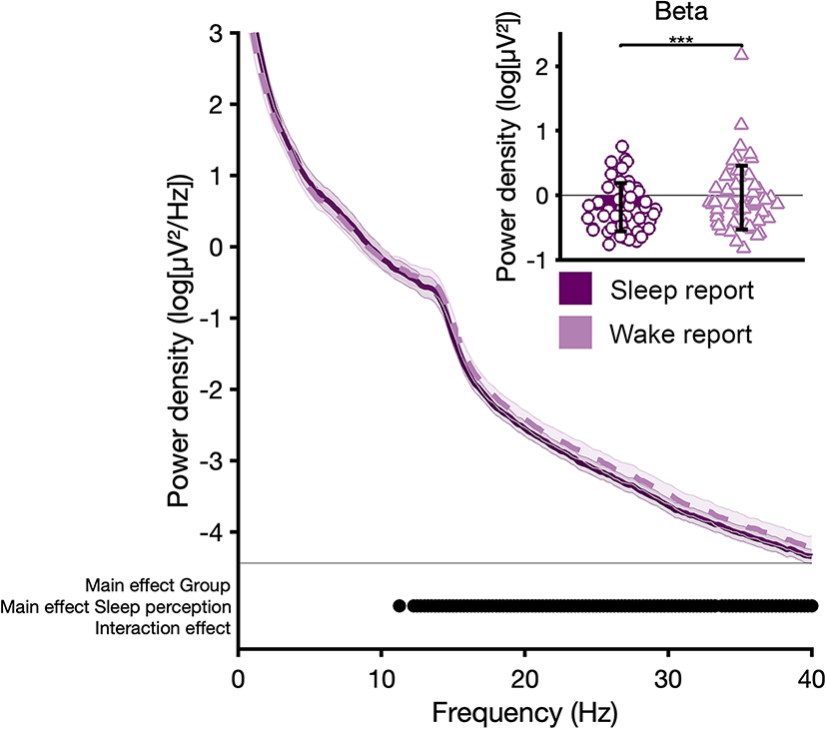
Spectral power as an average across the scalp during the 2 min block of NREM sleep preceding awakening during experimental night (95% CI). Significant differences between wake or sleep reports (groups collapsed) are indicated in the bottom plot for each 0.5 Hz bin (2-way ANOVA; *p* < 0.05). Inset figure shows increased beta band power prior to wake compared to sleep reports (paired samples t-test; *p* < 0.05). Single data points are plotted according to a kernel density estimation to visualize distribution shape.

The full EEG spectrum of NREM sleep prior to awakenings is presented in Fig. 6. ANOVAs revealed a significant effect (fast spindle and beta frequency range) for the repeated measures factor sleep-wake perception (*p* < 0.05), but no significant effect for the factor group (*p* > 0.05) and no significant interaction effect (*p* > 0.05). Elevated high frequency power predicted wake perception, without a group difference. We restricted the main analysis and interpretation to the EEG beta range.

### Effect sizes and bayesian estimates

**Fig. 7:**
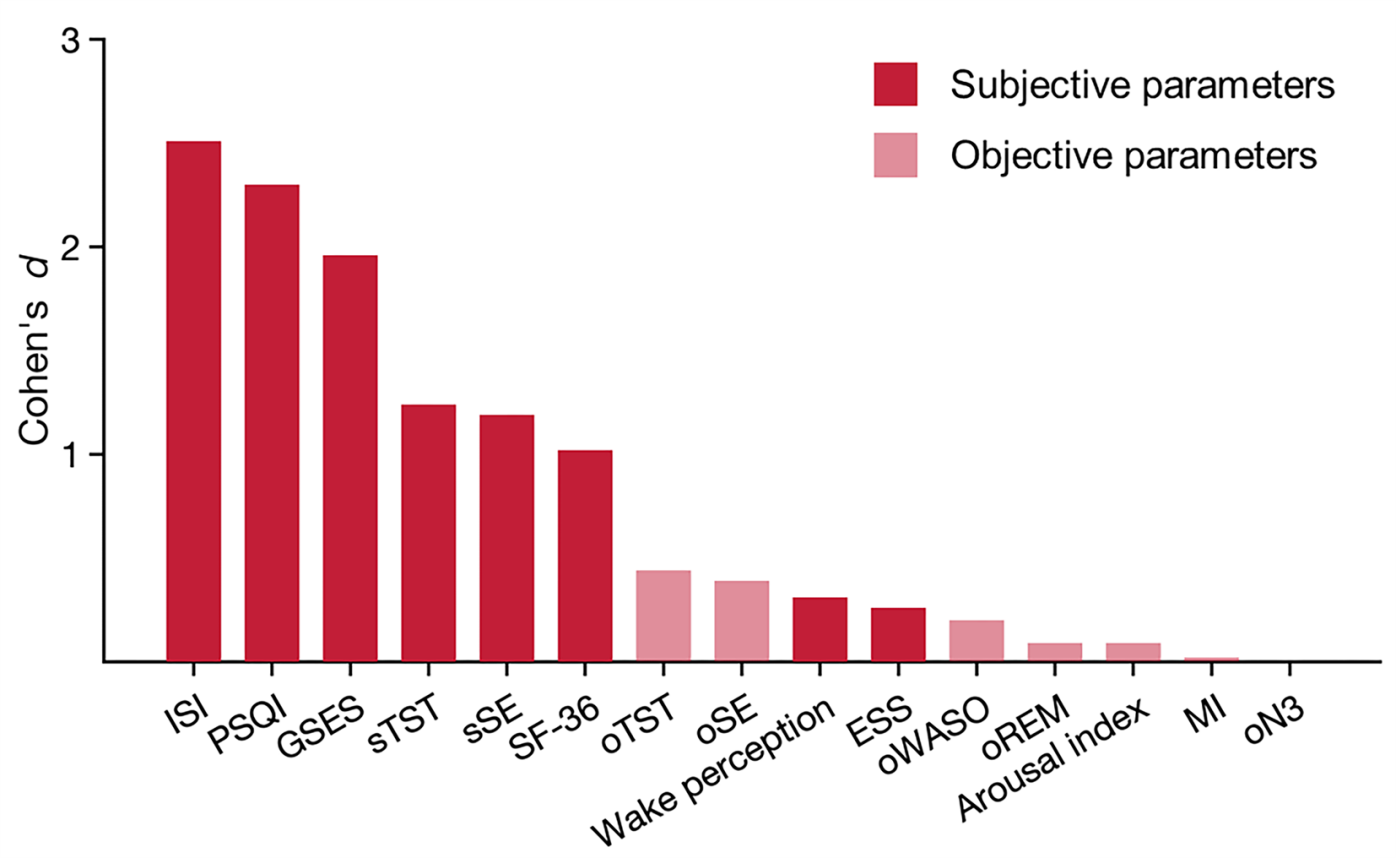
Effect sizes (ES) for group comparisons. Parameters refer to baseline, with the exception of wake perception that refers to the experimental night. Effect sizes are presented as positive values throughout, reflecting calculations in the direction of hypothesized effects. Insomnia severity index (ISI); Pittsburgh sleep quality index (PSQI); Glasgow sleep effort scale (GSES); Epworth sleepiness scale (ESS); Short form health survey, general health perception (SF-36); subjective total sleep time (sTST); subjective sleep efficacy (sSE); objective total sleep time (oTST); objective sleep efficacy (oSE); wake perception upon awakening during experimental nights; objective wake after sleep onset (oWASO); objective slow wave sleep (oN3); objective rapid eye movement sleep (oREM); arousal index; modulation index (MI). Parameters sorted by effect size. Differences are significant from ISI to sSE.

Figure 7 illustrates the effect sizes for the comparison of patients with insomnia disorder and healthy controls. Subjective parameters (darker red) exhibited large effect sizes in the expected direction, in comparison to objective parameters (lighter red), which showed small effect sizes. As exceptions, direct sleep-wake perception and daytime sleepiness as assessed by the Epworth Sleepiness Scale (both subjective parameters) demonstrate small to medium effect sizes. These findings suggest that measurements distant from actual night-time sleep show larger differences between the groups, indicating the potential role of cognitive and emotional processes in the pathophysiology of insomnia disorder.

To explore whether the absence of significant effects represents true evidence of no difference, we computed scaled JZS (Jeffreys–Zellner–Siow) Bayes Factors (BF) for selected parameters (JASP open-source statistics program, https://jasp-stats.org/). This analysis revealed that the evidence category for the null hypothesis is low (BF_01_ = 1.9 for objective WASO; BF_01_ = 1.2 for wake perception, %) to moderate (BF_01_ = 4.1 for modulation index, MI) (BF interpretation^31^. To adequately interpret these findings, we estimated sample sizes required to reach statistical significance for the respective parameters, revealing total sample sizes of 394 for objective WASO, 154 for wake perception, and 17’442 for MI (G*Power 3.1.9.2). To further put this in context, we calculated the JZS Bayes factor for the ISI (BF_01_ = 8.0 × 10^− 12^), indicative for an extreme level of evidence against the null hypothesis (or in other words, for a strong difference). Together this pattern of results indicates that differences in objective sleep parameters and direct wake perception might exist, but that they are most likely small and in evidence categories excessively smaller than those of the subjective ISI.

## Discussion

Patients with insomnia disorder, characterized by significant and clinically relevant subjective sleep complaints, did not differ from healthy controls in objective sleep parameters or direct sleep-wake perception. High frequency spectral power as an index of cortical arousal prior to awakening, but not standard measures of sleep, predicted the perception of wakefulness across groups, potentially related to wake-like activity during sleep. These results have potential implications for concepts of insomnia disorder and sleep-wake regulation, as well as future treatment developments.

Patients with substantial subjective sleep complaints did not differ from controls on objective sleep parameters. Patients reported sleep complaints at levels similar to previous studies, indicative of a representative clinical sample^15,32^. In contrast, patients did not differ from controls in objective sleep parameters. These findings are consistent with meta-analytic work showing minor differences in polysomnographic parameters between groups, such as total sleep time or wake after sleep onset (WASO)^8^. Our study adds to the literature by demonstrating no differences in fine-graded sleep parameters, including slow wave count, amplitude and duration, spectral slope in NREM and REM sleep, and phase-amplitude coupling between sleep slow oscillatory and spindle activity (modulation index). Moreover, we did not observe any differences in topographic analyses. Particularly, we do not replicate the finding of a pilot study on topographic differences^33^. These results suggest that sleep-wake regulatory systems are often intact in patients with insomnia disorder. This notion is further supported by the absence of increased daytime sleepiness (self-reports: current data; multiple sleep latency test, MSLT^34,35^, unchanged psychomotor vigilance^36^ - the two most sensitive indices for sleep loss - and unaffected glucose metabolism^37^ in patients with insomnia disorder.

Patients did not differ from controls in direct sleep-wake perception. Wake perception was frequent in 559 awakenings from NREM sleep, with 49.4% in controls and 59.4% in patients, without a group difference. Plots of single values and a low effect size suggest that we did not miss a relevant group difference (Fig. 4). Strengths of the current study include a high number of awakenings and a fully automatized protocol. The observed proportion of wake reports from NREM sleep falls within the broad variability documented in previous studies on controls and patients (70% vs. 88%^11^; 17.4% vs. 15.4%^14^; 55% vs. 96%^9^; 40% vs. 80%^12^; 18.6% vs. 22.4%^15^; 12.3% vs. 39.6%^13^). These findings suggest that the perception of wakefulness from NREM sleep is frequent and physiological and not limited to patients with insomnia disorder. Two studies observed more wake reports in patients than in controls^11,13^. Borkovec and colleagues preselected their sample of patients based on a sleep onset latency of 60 min or longer. Stephan and colleagues preselected 10 patients (“misperceptors”) who were characterized by extremely reduced subjective referred to objective total sleep time (“misperception index” < 60%). In our sample, none of the participants fulfilled this criterion, and in the larger sample of Benz and colleagues with 123 patients, 12 (9.8%) patients fulfilled the “misperceptor” criterion in a first night and only 3 (2.4%) patients in 2 consecutive nights^38^. Due to limited reliability and validity, both major diagnostic systems (DSM-5 and ICD-11) removed the subtype “misperception” (paradoxical insomnia) and focus on the diagnostic entity of insomnia disorder^1,3^.

Our study identified indices of increased cortical arousal during NREM sleep prior to wake reports across groups. More specifically, we observed increased EEG beta activity in the 2 min of NREM sleep prior to wake compared to sleep reports, without a group difference. EEG beta activity has been related to arousal processes, with a positive correlation to glucose metabolism in arousal systems during NREM sleep^19^. Increased EEG beta activity prior to reports of wake or lower sleep depth from NREM sleep has been reported in some serial-awakening studies^13^, but not in others^15^. Of note, standard polysomnographic parameters did not predict sleep-wake perception. The absence of a group effect suggests general mechanisms of sleep-wake perception that are not pathognomonic for insomnia disorder. Again, these findings suggest that fundamental sleep-wake regulatory systems are often intact in patients with insomnia disorder.

Our findings have limitations. The serial awakening protocol induced fragmented sleep, which might inflate wake reports in controls. Yet it appears unlikely that we missed a strong group effect in 559 awakenings. Patients reported worse sleep in the morning compared to controls, suggesting a representative character of the night. Further, participants might produce an answer after awakening that does not reflect their experience during sleep. However, prior work indicates that participants do report what was on their mind. This notion is supported by dream content studies in patients with parasomnia^39^, the incorporation of external stimuli during sleep with subsequent reports^40^, and the prediction of dream content through corresponding brain activity patterns^41^. Our study was not designed to test whether cognitive, emotional or behavioral factors (e.g., sleep-related anxiety) mediate the onset of the disorder. A major limitation of the current protocol is the lack of awakenings from REM sleep. Therefore, our findings cannot contribute to the REM sleep instability hypothesis^42–44^. We believe that characterizing REM sleep is of great interest and see NREM sleep studies as complementary towards a more comprehensive understanding of the complex perception of sleep and the pathophysiology of insomnia disorder.

Our results have potential conceptual and clinical implications. We propose that sleep-wake regulatory systems are often intact in patients with insomnia disorder. This perspective is also supported by a recent meta-analysis, which demonstrated an intact brain structure in patients with insomnia, without any significant associations between insomnia symptoms and cortical or subcortical volumes^45^. The clinical symptomatology appears to arise not from structural brain alterations, sleep loss and even not from direct sleep-wake perception, but through sustained cognitive (e.g., rumination, catastrophizing, generalization), emotional (e.g., anxiety) and behavioral mechanisms (e.g., extended bedtimes). The negative evaluation of sleep appears to arise over time with worries and anxiety towards sleep^38,46^. In their comprehensive review, Harvey and Tang argue that individuals are prone to gather evidence that confirms their core beliefs rather than evidence that challenges them (insomnia identity, “I am a bad sleeper”) and propose that memory for sleep may be biased by the worst and most recent night of sleep^6^. Future work is needed to determine whether patients in the diagnostic entity of insomnia disorder with objectively disrupted sleep have adverse health outcomes (for instance with regard to vigilance, cognitive performance, or glucose metabolism) and might benefit from different or additional interventions beyond CBT-I. As outlined in a conceptual review^47^, the subjective symptomatology of patients with insomnia disorder and sleep physiology represent at least partially independent dimensions. The systematic assessment of both might inform more nuanced diagnoses and tailored treatment strategies.

Our findings support CBT-I as the first-line treatment. While this recommendation is well established based on clinical studies, meta-analyses and guidelines^4^, our study further proposes an empirical basis for the indication of CBT-I. The current framework of largely intact sleep-wake systems suggests that hypnotics induce an unphysiological brain state. In fact, hypnotics might impair sleep-associated processes, like cortical plasticity in rodents (e.g., trazodone)^48^ and memory consolidation in humans (e.g., zolpidem)^49^. It would be relevant to further characterize the functional impact of different sleep medications. It appears important to inform patients and the public of often intact sleep regulatory systems. Advancements in the field might focus on empowering patients and health care teams, e.g. SLEEPexpert^50^ and other projects of adaptation, implementation and dissemination of CBT-I^51^. Future studies are also needed to determine the use of sleep measurements.

Our results have potential basic science implications. From an evolutionary perspective, sleep is a state of risk and maintaining some level of vigilance is vital^52^. Our findings are in line with concepts of fluid sleep-wake dynamics, for example local sleep in awake rats^16^ or the coexistence of wake- and sleep-like activity patterns in intracranial recordings in humans^17^. Further unraveling fluid sleep-wake dynamics represent major challenges for neurosciences and clinical development.

In conclusion, our study supports the notion that sleep-wake regulatory systems are often intact in patients with insomnia disorder. The complaints appear to not primarily emerge from sleep loss or direct wake perception, but potentially rather from sustained cognitive, emotional and behavioral mechanisms, which needs to be further tested. Our study furthermore highlights the need for novel concepts and measurements of sleep, possibly related to fluid sleep-wake regulation.

### Methods

#### Participants

Thirty patients with insomnia disorder according to DSM-5 criteria^1^ and 30 healthy controls completed the study protocol. Participants between 18 and 65 years were recruited via local advertisements. The diagnostic procedure followed guideline recommendations^4^ and included a clinical interview encompassing sleep and medical history, sleep questionnaires and diaries as well as a physical examination by experienced clinicians under the supervision of the last author. Participants were free of any acute mental, somatic or sleep disorder (except insomnia disorder for patients) according to clinical and sleep laboratory assessments. Participants were free of any central nervous system-active substances for at least two weeks prior to the study, did not smoke more than 5 cigarettes per day, did not excessively consume caffeine (> 500 mg or 5 cups coffee per day), and did not have an irregular sleep schedule (as documented by sleep diaries). Participants with an apnea hypopnea index (AHI) > 15 events per hour or a periodic leg movement index (PLMI) with arousal > 10 events per hour, as assessed during the first night in the sleep laboratory, were excluded. Specifically, two initially included patients with an AHI > 15 were excluded after the first night and referred for further clinical evaluation. Participants were reimbursed for participation. Power calculation for sample size estimation was done for the pre-registered parameter modulation index (G*Power 3.1.9.2; NCT04276064). Written informed consent was obtained from all participants prior to their participation. The study was conducted in accordance with the Declaration of Helsinki. The study was approved by the Ethics Committee of the Canton Bern (Project-ID: 2019–02426).

### Study design

**Fig. 8:**
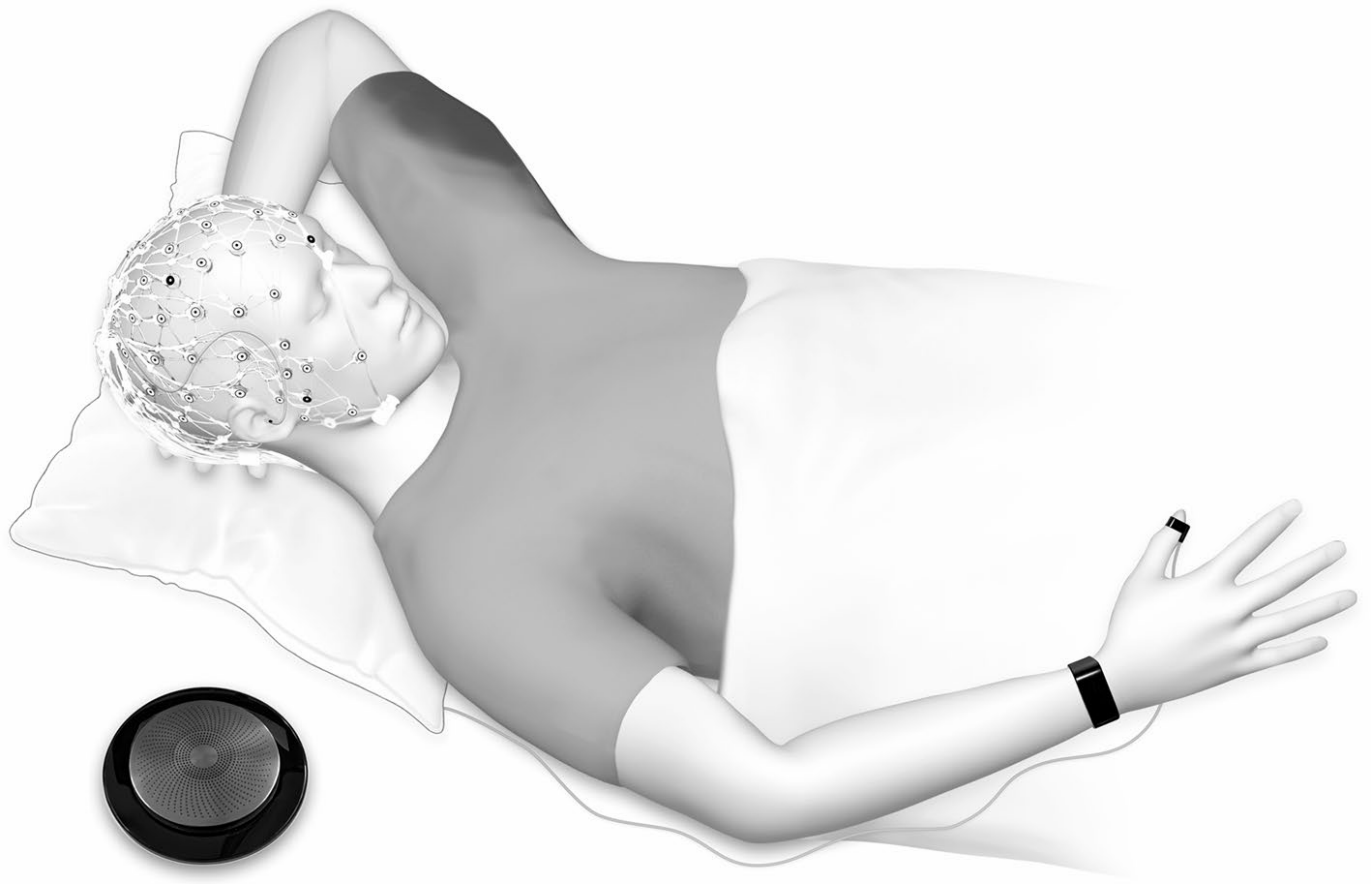
Illustration of a participant in the sleep laboratory setting. The setup included a high-density electroencephalogram (128 electrodes), a vibration bracelet (iBells) attached to the wrist serving as the awakening signal, a microswitch attached to thumb for initiation of interview questions, IP30 in-earphones (Diatec) for the transmission of interview questions, and an intercom for recording of the interview answers. The three-dimensional figure was created by the authors using the software MakeHuman 1.2.0, licensed under CC0 (https://static.makehumancommunity.org).

Participants were characterized clinically and tested across 3 nights in the sleep laboratory, including an adaptation (screening) night, a baseline night, and an experimental night with serial awakenings from NREM sleep. Participants reported on their sleep-wake perception through an automatized interview (set-up depicted in Fig. 8).

### Awakening procedure

**Fig. 9:**
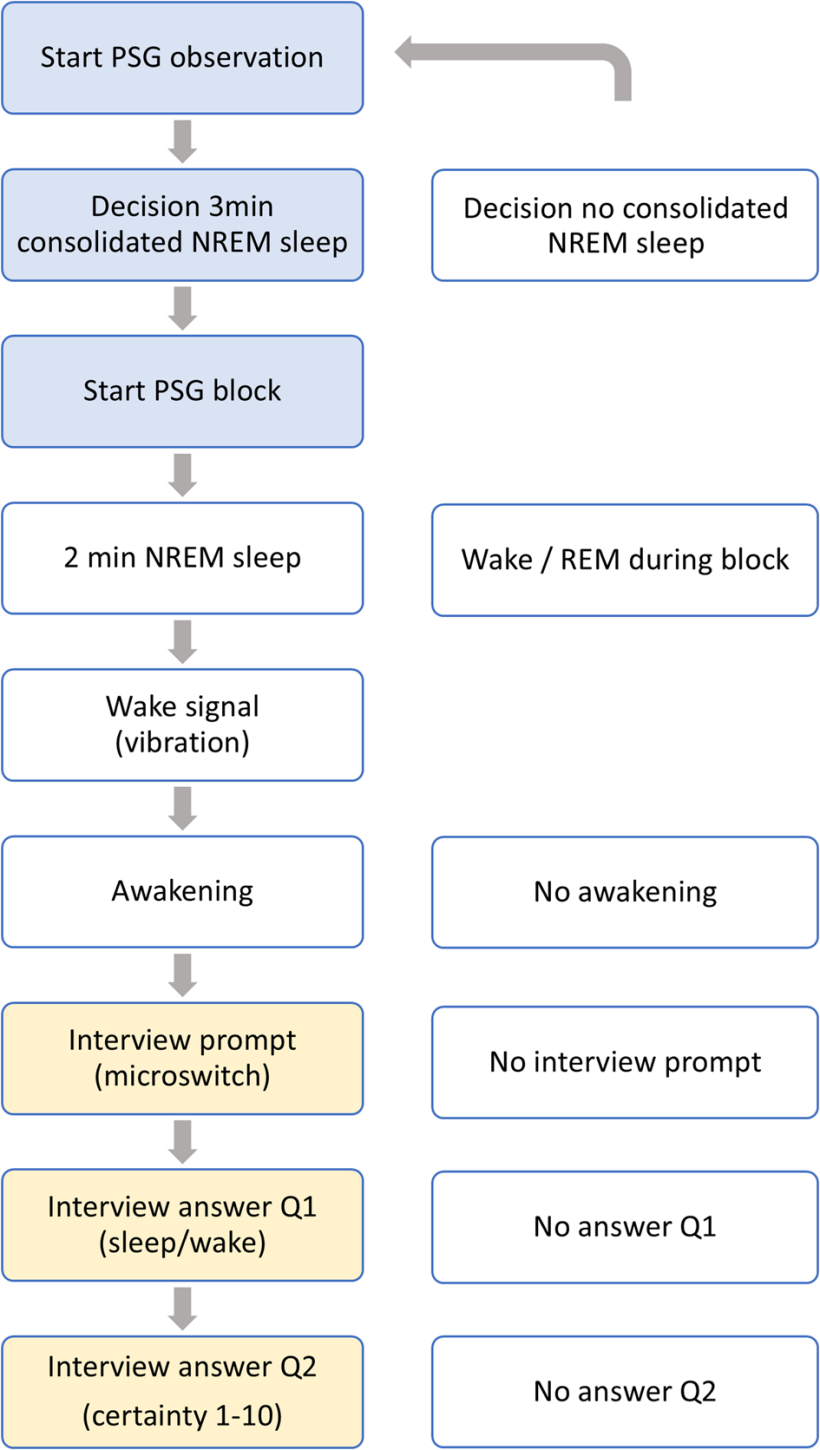
Flowchart of the awakening procedure and interview. The left side of the flowchart illustrates steps of a single awakening procedure from online NREM sleep detection to completion of the interview. This includes online detection of NREM sleep by a trained rater, countdown of 3 min of NREM sleep, the initiation of the awakening protocol starting with a set 2 min PSG block, an automatic wake signal via the vibration bracelet, awakening of the participant, interview prompt via microswitch by the participant, answer given to the first interview question (“Just now, were you asleep or awake?”), and an answer given to the second interview question (“How certain are you, from one to ten”), and completion of the interview. The right side of the flow-chart illustrates reasons for exclusion from primary analysis.

Participants were deliberately awakened up to 12 times out of NREM sleep during the experimental night and interviewed about their immediate subjective sleep-wake perception. Participants were informed that the interview could occur during wake or sleep (blinding). The awakening procedure included the following steps (Fig. 9): online raters observed PSG sleep and awaited a period of 3 min of consolidated NREM sleep. In case of arousals or shifts to wake or REM sleep, the next period of 3 min of NREM sleep was awaited. After 3 min of NREM sleep, the raters initiated a fully automatized awakening and interview protocol, which consisted of the following steps: a 2 min block with polysomnographic monitoring, followed by the awakening signal applied through a vibration bracelet (iBells, Germany) affixed to the wrist of the participant. A maximum time window of 45 s was set to react to the awakening signal. Participants were instructed to double-click a microswitch button (affixed to the thumb or index finger)^15^ of the right hand upon feeling the awakening signal. A double-click initiated the delivery of pre-recorded interview questions over in-ear phones (IP30, Diatec). Answers were recorded via an intercom (Jabra Speak, Jabra, Denmark). The automated awakening procedure allowed for standardization and minimal disruption of the participant. Of note, participants did not have access to watches or phones during the night. The awakening procedures were conducted by 3 raters trained in online electroencephalography (EEG) scoring by an experienced rater. Raters showed comparable online sleep scoring.

### Interview

The interview was adapted from Weigand^10^ as used by Feige and colleagues^15^ and consisted of the following pre-recorded questions: (1) “Just now, were you awake or asleep?”, with the instruction to answer “wake” or “sleep”. (2) “How certain are you, from one to ten?”, with the instruction to answer 1 (low certainty) to 10 (high certainty). Participants advanced through the interview questions by using the microswitch to move from one question to the next. This process was practiced prior to sleep. A first awakening protocol was conducted during wakefulness shortly after lights-off to ensure functioning and blinding. Participants were instructed to speak their answer aloud into the room, where the answers were picked up by an intercom, recorded and later transcribed. Participants were awakened from consolidated NREM sleep, validated offline for inclusion into the final analysis. Answers were rated as sleep or wake.

### Sleep recordings

Electroencephalography (EEG) was recorded throughout each of the nights using a high-density EEG cap (128 electrodes; MicroCEL, EGI Philips, USA) as well as electrocardiogram, connected to a Net Amps 400 EEG amplifier (Electrical Geodesics, Inc., USA). The time in bed was set to eight hours, adapted to the individual rhythm. The adaptation night, used to familiarize the participant to the sleep laboratory environment and to test for organic sleep disorders, additionally included monitoring of electrooculography (EOG) electrodes, 2 submental electromyography (EMG) electrodes, recording of abdominal and thoracic effort, nasal airflow, oximetry and bilateral tibialis anterior EMG. The EEG was recorded at a sampling rate of 500 Hz, referenced to channel Cz, and was pre-processed using MATLAB (R2023a, MathWorks Inc., Natick, MA, USA), the EEGLAB toolbox^53^ http://sccn.ucsd.edu/eeglab/) and the FieldTrip analysis toolbox^54^. Polysomnographic recordings were visually scored off-line by an experienced rater according to standard criteria^55^, blinded to the group (patients or controls). The EEG data used for analyses was first down-sampled to 200 Hz. The data was then notch filtered (between 47 and 53 Hz) to remove line noise and bandpass filtered between 0.1 and 95 Hz. Further preprocessing was carried out using the PREP pipeline^56^, involving line noise removal, re-referenced relative to an estimated true average reference after removing contamination by bad channels, and detection and interpolation of bad channel based on extreme amplitudes, lack of correlation with any other channel, lack of predictability by other channels and unusually high frequency noise. Rejected channels were interpolated using spherical interpolation^56^. Remaining artifactual sections of the data were removed in a semi-automatic fashion. After removing wake EEG data, bad periods (in 30 s bins) were tagged based on EEG band power for further visual inspection. This pipeline was based on an approach published by Mariani and colleagues^57^. On average across subjects, 97.2% (± 3.2) of frames during sleep was retained for further processing and 11.1% (± 10.4) of the channels were rejected and interpolated. The following polysomnographic parameters of sleep continuity and architecture were assessed for the baseline night: Total sleep time (TST), defined as the time spent in N1, N2, N3 or REM sleep after sleep onset; sleep efficiency (SE), defined as the ratio of TST to time in bed × 100%; sleep-onset latency (SOL), defined as the period between “lights off” and the first 30 s epoch of N2, N3 or REM sleep; REM sleep latency, defined as the period between sleep onset and the occurrence of the first 30-s epoch of REM sleep; wake after sleep onset (WASO), defined as the wake time from sleep onset to “lights on”; time spent in sleep stages N1, N2, N3 and REM sleep, as a percentage of TST; arousal index, defined as the number of arousals per hour of sleep.

Spectral power density was calculated using Welch’s method, on 10 overlapping 4-s sub-epochs per 30-s time bin using a Hanning window. 30-s bins containing low frequency (0.75–4.5 Hz) and high frequency (20 to 40 Hz) artifacts were additionally tagged and disregarded from further spectral analyses. Low and high frequency artifacts were defined as power values 2 x moving median of 15 × 30 s epochs, as well as being above 10 or 3000 µV^2^/Hz respectively. Outlier channels were excluded before calculating averages across the scalp. Outlier channels were defined as elements more than 3 scaled mean absolute deviations from the median. Furthermore, all analyses were restricted to 95 channels overlaying the scalp, removing peripheral channels that are susceptible to artifacts^58^. The spectral power values were log-transformed (base e), and all subsequent steps including statistical analysis were performed on these logarithmic values. Slow waves were detected during N2 and N3 as previously reported^59^. The EEG was bandpass filtered between 1 and 2 Hz. The count, peak amplitude and density (count/min) was calculated for detected slow waves per channel and averaged across the scalp. To measure the strength of coupling between phase of slow waves and spindle peak amplitude (modulation index, MI) was calculated in 30 s bins during N2 and N3 for the slow oscillation (0.5–1 Hz) and fast spindle band (12–16 Hz, *21*). Finally, we estimated the spectral slope from a linear regression fit to the power spectrum in log-log space from 30 to 45 Hz^60,61^, as an index of non-oscillatory components of the power spectrum.

To explore global and local spectral changes across the scalp, the topographic distribution across derivations for log transformed spectral densities were analysed for the following frequency bands: SWA 0.75–4.5 Hz, theta 4.5–9 Hz, alpha 9–15 Hz, slow spindle activity 9–12 Hz, fast spindle activity 12–15 Hz, and beta 20–40 Hz. Group differences were analyzed using t-tests for independent samples, corrected for multiple comparisons across channels by false discovery rate (FDR^30^.

### Statistics

MATLAB (R2023a, MathWorks Inc., Natick, MA, USA) was used for statistical analyses. Data are reported as means ± standard deviations, if not indicated otherwise. Independent sample t-tests or chi-square tests were used to compare demographic, clinical and sleep characteristics between patients and controls. Analyses of variance (ANOVA) were calculated to explore the effects of group (insomnia vs. control) and sleep perception (sleep vs. wake) during the experimental night. Greenhouse-Geisser correction was applied in the case of lack of sphericity in the ANOVAs based on a Mauchly test. Power calculation was done for the pre-registered parameter modulation index (G*Power 3.1.9.2). To estimate effect sizes, Cohen’s d values were calculated for pairwise comparisons (small < 0.5; medium: ≥ 0.5 and < 0.8; large: ≥ 0.8), while partial eta square values (ηp2) were calculated for ANOVAs (small: <0.06; medium: ≥0.06 and < 0.14; large: ≥0.14). To visualize distribution shape, single data points were horizontally dispersed based on a kernel density estimation. The level of statistical significance was set at *p* < 0.05 for all analyses (two-tailed).

## Electronic supplementary material

Below is the link to the electronic supplementary material.

Supplementary Material 1

## Data Availability

Data is provided within the manuscript or supplementary information files.
