# Supplementary material for: Multimodal assessment of sleep-wake perception in insomnia disorder
Source: Sci Rep. 2025 Jun 5;15:19328. doi: 10.1038/s41598-025-00995-3 (PMC12141479; doi:10.1038/s41598-025-00995-3)
Supplement: Supplementary file 1 — Supplementary Material 1 [file 41598_2025_995_MOESM1_ESM.pdf]

Supplementary Table 1. Baseline statistics

	Healthy controls (n=30)	Insomnia patients (n=30)	p-value	ES
Polysomnography				
Total sleep time (minutes)	413.7 ± 45.7	390.0 ± 59.9	p = 0.090	0.45
Sleep efficiency (%)	86.2 ± 9.6	81.9 ± 12.3	p = 0.130	0.40
Sleep latency (minutes)	16.9 ± 15.5	22.2 ± 18.1	p = 0.230	0.31
REM sleep latency (minutes)	79.4 ± 29.2	79.8 ± 31.4	p = 0.966	0.01
Wake after sleep onset (minutes)	45.6 ± 45.1	55.1 ± 49.4	p = 0.440	0.20
N1 (%, total sleep time)	14.3 ± 6.9	17.4 ± 10.6	p = 0.200	0.33
N2 (%, total sleep time)	52.4 ± 8.0	48.9 ± 7.1	p = 0.077	0.47
N3 (%, total sleep time)	14.0 ± 7.7	13.9 ± 9.5	p = 0.975	0.01
REM sleep (%, total sleep time)	19.3 ± 6.9	19.9 ± 5.7	p = 0.723	0.09
Arousal index (arousal/hour)	11.6 ± 4.2	11.9 ± 3.4	p = 0.723	0.09
Sleep microstructure				
Spectral slope NREM	- 2.5 ± 1.4	- 2.2 ± 0.4	p = 0.257	0.30
Spectral slope REM	- 2.5 ± 1.0	- 2.3 ± 0.6	p = 0.515	0.17
Slow wave count	2956.9 ± 489.5	2691.6 ± 716.3	p = 0.099	0.43
Slow wave amplitude	21.5 ± 5.9	21.4 ± 7.2	p = 0.944	0.02
Slow wave duration	0.6 ± 0.01	0.6 ± 0.02	p = 0.124	0.40
Modulation index	(4.2 ± 0.6) $\times 10^{-3}$	(4.2 ± 0.4) $\times 10^{-3}$	p = 0.916	0.03
Subjective sleep parameters				
Total sleep time (minutes)	444.0 ± 36	390 ± 54	**p < 0.001**	1.25
Sleep efficiency (%)	90.5 ± 6.6	80.0 ± 10.6	**p < 0.001**	1.19
Sleep quality	5.2 ± 1.3	4.3 ± 1.4	**p = 0.020**	0.62
Sleep latency (minutes)	20.0 ± 15.0	30.7 ± 21.8	**p = 0.034**	0.58
Daytime tiredness	4.6 ± 1.7	2.7 ± 1.7	**p < 0.001**	1.13
Concentration	4.9 ± 1.6	3.6 ± 1.9	**p = 0.006**	0.80
Mood	5.6 ± 1.0	4.4 ± 1.6	**p = 0.001**	0.90
Feeling of restoration	5.2 ± 1.2	4.1 ± 1.5	**p = 0.003**	0.81

Data represents means ± standard deviations. Percentages where indicated. Independent sample t-tests, ES, effect size, Cohen's *d*. Significant p-values are written in bold.
